# The possible mechanisms of ferroptosis in sepsis-associated acquired weakness

**DOI:** 10.3389/fphys.2024.1380992

**Published:** 2024-03-27

**Authors:** Jun Yang, Caihong Yan, Shaolin Chen, Min Li, Yanmei Miao, Xinglong Ma, Junfa Zeng, Peng Xie

**Affiliations:** ^1^ Department of Critical Care Medicine of the Third Affiliated Hospital (The First People’s Hospital of Zunyi), Zunyi Medical University, Zunyi, China; ^2^ Department of Critical Care Medicine, The Second Affiliated Hospital, Hengyang Medical School, University of South China, Hengyang, China; ^3^ Department of Nursing of Affiliated Hospital, Zunyi Medical University, Zunyi, China

**Keywords:** sepsis-associated acquired weakness, ferroptosis, iron, lipid metabolic disorders, Sxc^−^, p53

## Abstract

Sepsis is a life-threatening organ dysfunction caused by a dysregulated host response to infection, and its morbidity and mortality rates are increasing annually. It is an independent risk factor for intensive care unit-acquired weakness (ICU-AW), which is a common complication of patients in ICU. This situation is also known as sepsis-associated acquired weakness (SAW), and it can be a complication in more than 60% of patients with sepsis. The outcomes of SAW are often prolonged mechanical ventilation, extended hospital stays, and increased morbidity and mortality of patients in ICUs. The pathogenesis of SAW is unclear, and an effective clinical treatment is not available. Ferroptosis is an iron-dependent type of cell death with unique morphological, biochemical, and genetic features. Unlike other forms of cell death such as autophagy, apoptosis, and necrosis, ferroptosis is primarily driven by lipid peroxidation. Cells undergo ferroptosis during sepsis, which further enhances the inflammatory response. This process leads to increased cell death, as well as multi-organ dysfunction and failure. Recently, there have been sporadic reports suggesting that SAW is associated with ferroptosis, but the exact pathophysiological mechanisms remain unclear. Therefore, we reviewed the possible pathogenesis of ferroptosis that leads to SAW and offer new strategies to prevent and treat SAW.

## Introduction

Sepsis is a life-threatening dysfunction caused by a dysregulated host response to infection. The World Health Organization categorizes it as a global health priority (Cecconi et al., 2018; Evans et al., 2021). Despite many advances in the clinical management of sepsis, treatments are limited to symptomatic treatments, such as fluid resuscitation and organ protective support (Evans et al., 2021). The prevalence of sepsis and septic shock has increased annually since it was initially defined. Among 49 million patients with sepsis in 2017, 11 million succumbed to avoidable deaths that accounted for almost 20% of all-cause deaths and the prevalence was higher in females than in males (World Health Organization, 2020). The mortality rate of sepsis in intensive care unit (ICU) is 42%, which is very high (Crawford et al., 2023).

Intensive care unit-acquired weakness (ICU-AW) is a frequent complication among critically ill patients. It is mainly characterized by generalized symmetrical decrease in proximal limb muscle tone and diminished or normal deep tendon reflexes, which are often cumulative in limb and respiratory muscles (Stevens et al., 2009; Latronico et al., 2017). Globally, 13–20 million patients annually require treatment in ICU and >1 million of them develop ICU-AW (Fan et al., 2014). The main etiologies of ICU-AW are sepsis, mechanical ventilation, long-term immobilization, hyperglycemia, advanced age, and malnutrition (Herridge et al., 2011; Jolley et al., 2016; Ozdemir et al., 2022). Sepsis is an independent risk factor for ICU-AW, affecting over 60% of sepsis patients (Latronico et al., 2017; Mitobe et al., 2019; Bellaver et al., 2023; Takahashi et al., 2023), and this condition is also known as sepsis-associated acquired weakness (SAW). The pathogenesis of SAW remains unclear and an effective clinical treatment does not exist. Ferroptosis is closely associated with sepsis, such as lipid peroxidation, reactive oxygen species (ROS), and cellular ion metabolism (Huang et al., 2023). Ferroptosis is involved in skeletal muscle atrophy and sarcopenia, and sepsis can cause acquired muscle weakness (Ikeda et al., 2016; Huang et al., 2021). Inflammation inhibits the expression of the ferroptosis biomarker cystine transporter solute carrier family 7 member 11 (SLC7A11) and GPX4 synthesis is blocked, thus promoting the occurrence of ferroptosis (Wang Y. et al., 2023; Wang Z. et al., 2023). However, whether ferroptosis is involved in the development of SAW is unclear. Therefore, we reviewed the mechanisms of ferroptosis that might be involved in SAW to generate new strategies that could prevent and treat SAW.

Ferroptosis is a unique form of iron-dependent non-apoptotic cell death that is morphologically, biochemically, and genetically distinct from apoptosis, necrosis, and autophagy (Dixon et al., 2012). The essence is that intracellular ROS and hydrogen peroxide (H_2_O_2_) produce lipid peroxides due to the action of iron and oxidized lipid membranes with polyunsaturated fatty acids (PUFAs). This causes membrane damage followed by cell death. The intracellular cystine/glutamic acid reverse transporter (SXc^−^), glutathione, and glutathione peroxidase 4 (GPX4) interact under physiological conditions to maintain homeostasis in cells and organisms (Dixon et al., 2012; Hassannia et al., 2019; Jiang et al., 2021). Mitochondria are not only involved in energy supply but also produce ROS, H_2_0_2_ in the process. In general, mitochondrial antioxidant enzymes, such as superoxide dismutase (SOD), glutathione peroxidase (GPX), catalase, etc., can scavenge excess ROS and H_2_0_2_ produced during metabolism, maintain mitochondrial homeostasis, and protect cellular activity (Paplou et al., 2021). Furthermore, the mitochondria-targeted antioxidant MitoTEMPO blocked adriamycin-induced cardiac ferroptosis in mice, providing strong *in vivo* evidence for a link between mitochondria and ferroptosis (Fang et al., 2019). Ferroptosis is involved in the development of various diseases such as sepsis, tumors, neurological disease, and cardiomyopathies (Qiu et al., 2020). When bacterial infections result in sepsis, bacteria rely on iron for growth and thus stimulate intracellular iron shedding and release. Moreover, bacteria enhance the production of ROS and unsaturated fatty acids that serve as additional resources for ferroptosis and as bacterial nutrients. This subsequently exacerbates infection, leading to multi-organ dysfunction and ICU-AW (Latronico et al., 2017; Lei et al., 2022; Ma et al., 2022).

### Possible mechanisms of ferroptosis leading to SAW

Ferroptosis plays important roles in sepsis-induced lung and renal injury, cardiomyopathy (Li et al., 2020; Wang et al., 2020; Li et al., 2022; Qiongyue et al., 2022; Bayır et al., 2023; Fang et al., 2023) and in the pathological process of sarcopenia and skeletal muscle atrophy (Reardon and Allen, 2009; Huang et al., 2021). Ferroptosis in skeletal muscle cells might be involved in the development of SAW, and the mechanism might be associated with disordered iron metabolism, lipid peroxidation, and oxidative stress that inhibit GPX4 expression and promote ferroptosis in skeletal muscle cells (Li et al., 2023). We previously found that the AMP-activated protein kinase-peroxisome proliferator-activated receptor γ coactivator-1α-nicotinamide adenine dinucleotide (NAD)-dependent deacetylase sirtuin-3 (SIRT3) signaling pathway is involved in SAW development (Wang, 2021) and that SIRT3 upregulation inhibits p53-mediated ferroptosis (Jin et al., 2021). SIRT3 is mainly located on mitochondria (Zhou et al., 2022), which are impaired in muscle in sepsis patients, interfering with muscle function and metabolism (Fredriksson et al., 2008; Friedrich et al., 2015; Leduc-Gaudet et al., 2020). Mitochondrial dysfunction can also be considered a “catalyst” for skeletal muscle atrophy. Mitochondrial dysfunction is not limited to muscle atrophy, but also triggers a cascade of deleterious events, including impaired energy metabolism, increased oxidative stress, and ferroptosis (Owen et al., 2019).

It has also been found that in a mouse model of sepsis, mitochondrial ultrastructural features manifested as smaller mitochondria, increased membrane density, reduced or absent cristae and rupture of the outer membrane, providing additional evidence for iron-induced cell death in skeletal muscle cells of septic mice (Li et al., 2023). In addition, the researchers identified a set of genes associated with both mitochondrial dysfunction and ferroptosis in sepsis-affected skeletal muscle and subjected the muscle tissue to immunoblotting analyses that revealed significantly increased levels of ferroptosis marker proteins (Sheng et al., 2024). The morphological features of ferroptosis are mainly high mitochondrial membrane density, reduced volume and rupture of the outer membrane (Jiang et al., 2021). Cells undergoing ferroptosis are immunogenic and can amplify the inflammatory response, causing more cell death (Martin-Sanchez et al., 2017). We speculated that the common mechanism of ferroptosis leading to sepsis and muscle atrophy are closely associated with the pathogenesis of SAW. Therefore, this article focuses on relevant mechanisms that may contribute to the development of SAW, such as iron and lipid metabolic disorders, SXc^−^, and p53.

#### Iron metabolism disorders

**FIGURE 1:**
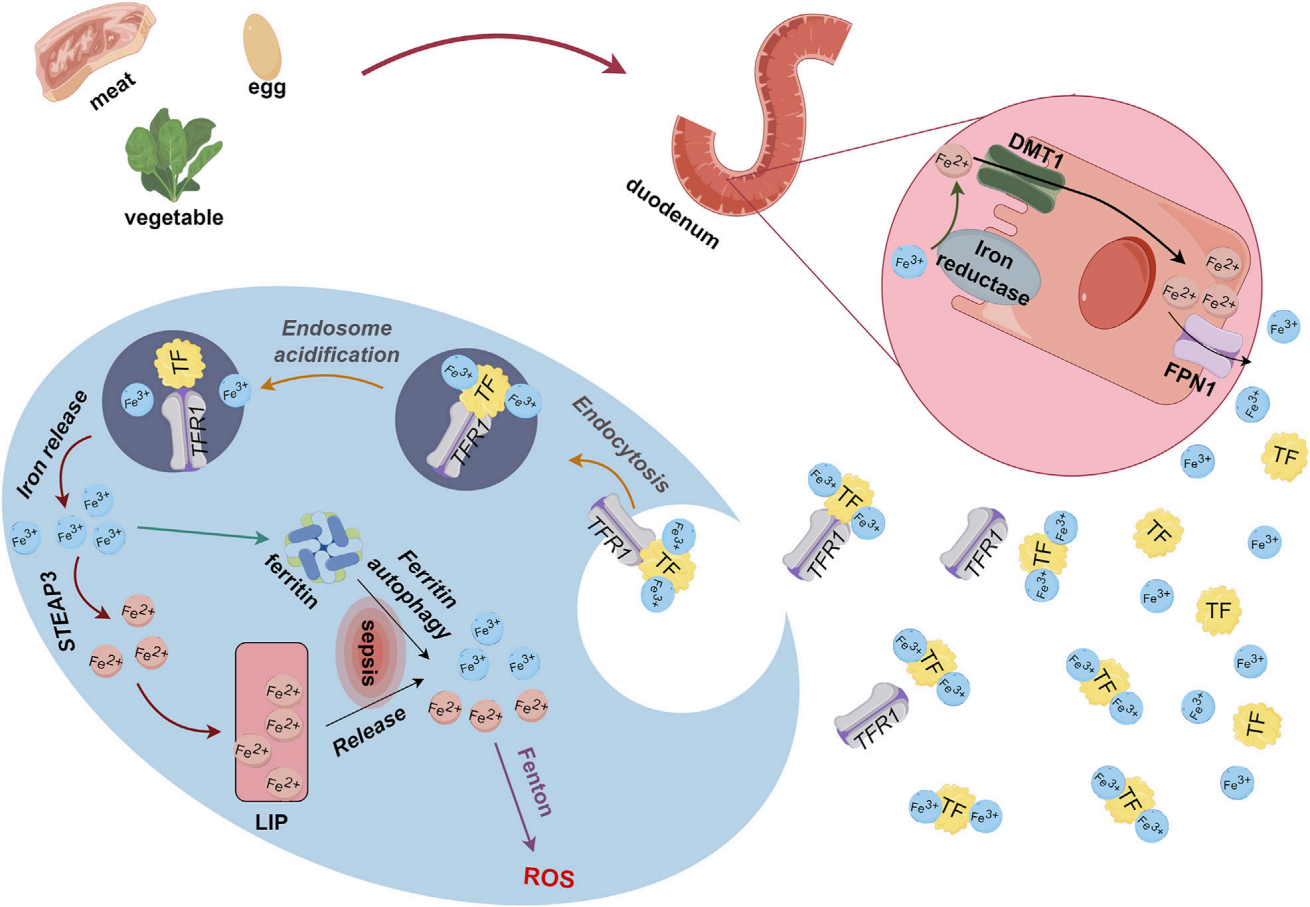
Metabolism of dietary iron derived from food. Ferric reductase converts Fe^3+^ absorbed through the duodenum to Fe^2+^, which binds to DMT1 for transport to enterocyte apical membranes. Endocytosis causes Fe^2+^ and FPN1 to enter blood circulation, and free Fe^2+^ binds to TF as TF-Fe^3+^. Thereafter, TFR1 transports Fe^3+^ through endocytosis into cells where some iron is stored as ferritin and some is converted to Fe^2+^ by STEAP3 and enters LIP. DMT1, divalent metal-ion transporter 1; Fe, iron; LIP, labile iron pool; STEAP3, 6-transmembrane epithelial antigen of the prostate 3; TF, transferrin; TFR1, transferrin receptor 1.

Iron is essential for life as it plays important roles in physiological and redox reactions, as well as DNA synthesis (Liu et al., 2021). Iron concentrations that exceed a threshold maintained by homeostatic mechanisms can trigger increased ROS production, oxidative stress, ferroptosis, activation of pro-inflammatory signaling pathways, and other toxic responses (Ward and Cloonan, 2019; Dutt et al., 2022). Most iron enters the body through dietary iron intake. Dietary iron is usually regarded as heme or non-heme and most of it enters the body in the ferric (Fe^3+^) form, which ferric reductase reduces to the ferrous (Fe^2+^) form. After binding to divalent metal-ion transporter 1 (DMT1) in the intestinal lumen, Fe^2+^ crosses the brush border membrane to reach the apical membranes of enterocytes. Ferroportin 1 (FPN1) transports Fe^2+^ across the basolateral membrane and into the blood circulation by cytosis and binds to plasma transferrin (TF) to form TF-Fe^3+^ complexes that enter cells through endocytosis by transferrin receptor 1 (TFR1). Some Fe^3+^ is stored in ferritin. Excess Fe^3+^ converted to Fe^2+^ by ferric reductases (6-transmembrane epithelial antigen of the prostate 3, STEAP3) enters the labile iron pool (LIP) (Anderson and Frazer, 2017) (Figure 1). Intracellular iron is normally in dynamic equilibrium and is used to meet the normal metabolic demands of organisms. However, due to repeated blood sampling or blood losses, anemia of inflammation (AI), along with blood transfusions and intravenous iron supplementation, ICU patients face challenges in maintaining a dynamic iron balance (Grange et al., 2023).

Under pathological conditions, proteins associated with iron and ferritin metabolism can become dysfunctional and augment biomarkers such as iron and ferritin that are associated with inflammation. Ferritin autophagy and iron deposition during inflammatory processes (Liu et al., 2021; Hamilton et al., 2023) result in excessive intracellular accumulation of Fe^3+^ and Fe^2+^. This excessive Fe^3+^ and Fe^2+^ accumulation thereby leads to ROS accumulation via the Fenton reaction, which generates lipid peroxides (PL-PUFA-OOH) that lead to ferroptosis (Xing et al., 2022). In addition, excessive iron in skeletal muscle induces skeletal muscle atrophy and oxidative stress (Reardon and Allen, 2009; Ikeda et al., 2016), such as the loss of skeletal muscle mass in aged rats (Huang et al., 2021). Both sepsis and skeletal muscle atrophy can lead to disordered iron metabolism, which is the mechanism of ferroptosis. We thus postulated that disrupted iron metabolism is one explanation for ferroptosis in SAW.

#### Disordered lipid metabolism

Lipids considerably impact cellular structure and functioning, including their involvement in biofilm composition and signaling processes. Polyunsaturated fatty acids are important components of the lipid bilayer in cell membranes, and ferroptosis is characterized by the generation of PL-PUFA-OOH, which is considered as the main executer of ferroptosis (Jiang et al., 2021). Free PUFAs synthesize lipids via synthases and integrate with phospholipids to form PUFA-PL to maintain cell membrane stability (Welch et al., 2022). Physiologically, cytochrome P450 oxidoreductase (POR) is located in the endoplasmic reticulum (Koppula et al., 2021; Rojas Velazquez et al., 2022). The electrons of nicotinamide dinucleotide phosphate hydrogen (NAD(P)H) are transferred to downstream CYP450 (Yan et al., 2021) via POR; however, some of these electrons react with oxygen during transfer, which results in H_2_O_2_ production. The subsequent Fenton reaction between H_2_O_2_ and Fe^2+^ leads to the generation of free hydroxyl radicals (-OH) that oxidatively react with PUFAs to yield PL-PUFA. Under the influence of Fe^2+^, PL-PUFA transforms into toxic PL-PUFA-OOH (Liang et al., 2022). Interaction between PL-PUFA-OOH and reduced glutathione (GSH) leads to their reduction via GPX4 to yield PUFA phospholipid alcohols (PL-PUFA-OH) and oxidized glutathione (GSSG). This enzymatic process helps preserve cell membrane integrity.

**FIGURE 2:**
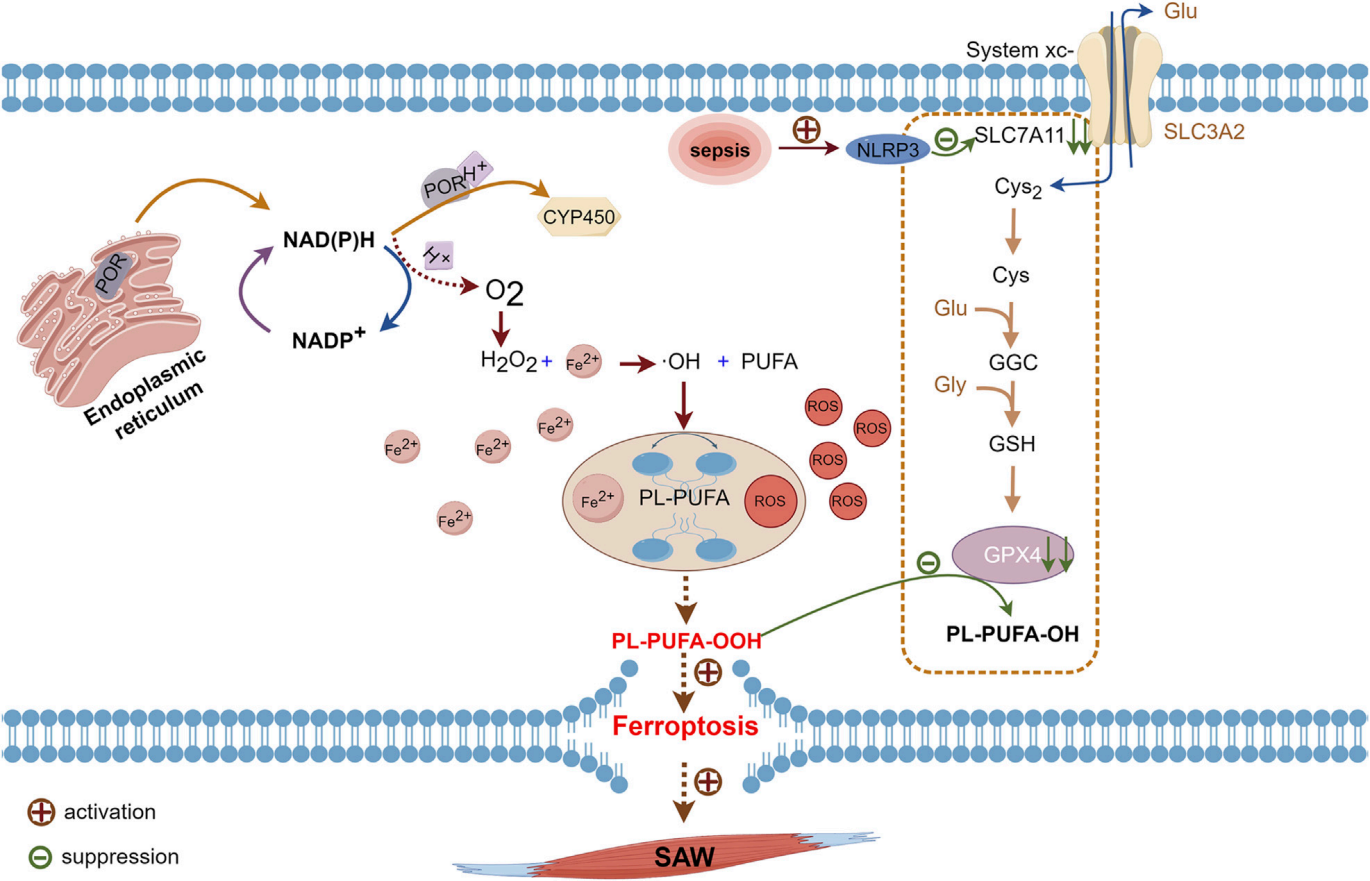
Pathways of PUFAs involved in ferroptosis. Cytochrome P450 oxidoreductase on endoplasmic reticulum transfers electrons from NAD(P)H to CYP450 while some NAD(P)H transfers electrons to oxygen and produces H_2_O_2_. Hydrogen peroxide reacts with Fe^2+^ in the Fenton reaction to form -OH, which reacts with oxidized PUFAs to form PL-PUFA, which ROS and Fe^2+^ will convert to PL-PUFA-OOH. Intracellular GPX4 under physiological conditions will reduce PL-PUFA-OOH to PL-PUFA-OH. During sepsis, activated NLRP3 inhibits the expression of SLC7A11, which hinders cystine uptake, leaving insufficient raw material for GPX4 synthesis. Due to reduced levels of GPX4 synthesis, PL-PUFA-OOH cannot be reduced, which leads to ferroptosis. -OH, hydroxyl radical; CYP450, cytochrome P450; GPX4, glutathione, and glutathione peroxidase 4; H_2_O_2_, hydrogen peroxide; NAD(P)H, nicotinamide dinucleotide phosphate hydrogen; NLRP3, nucleotide-binding domain, leucine-rich–containing family, pyrin domain–containing-3; PL-PUFA, polyunsaturated-fatty-acid-containing phospholipid; PL-PUFA-OH, polyunsaturated-fatty-acid-containing oxidative phospholipid; PL-PUFA-OOH, polyunsaturated-fatty-acid-containing-phospholipid hydroperoxides; ROS, reactive oxygen species; SLC7A11, cystine transporter solute carrier family 7 member 11.

Pathological conditions such as sepsis and myasthenia, disrupt the equilibrium described above. That is, excessive -OH production and GPX4 depletion prevents the continued reduction of PL-PUFA-OOH and leads to oxidative rupture of the cell membrane (Ursini and Maiorino, 2020; Yan et al., 2021). Meanwhile, the expression of light chain SLC7A11 is suppressed by the activation of the nucleotide-binding domain, leucine-rich–containing family, pyrin domain–containing-3 (NLRP3) inflammasomes during sepsis (Liu et al., 2022; Wang C. et al., 2023). Downstream GPX4 levels are decreased, and inhibiting this pathway accelerates the onset of ferroptosis (Wang Y. et al., 2023; Wang Z. et al., 2023). Inflammation also contributes to the production and accumulation of ROS in the skeletal muscle, and this is a major characteristic of skeletal muscle atrophy (Eshima et al., 2023). Accumulated ROS leads to increased PL-PUFA-OOH level and inhibited GPX4 activity, which results in ferroptosis (Hassannia et al., 2019), thus exacerbating the development of sepsis. This process also accelerates GPX4 depletion, which facilitates ferroptosis (Figure 2). Thus, ferroptosis induced by disordered lipid metabolism disorders might be an additional pathogenetic mechanism of SAW.

#### System Xc^−^-GSH-GPX4 pathway

**FIGURE 3:**
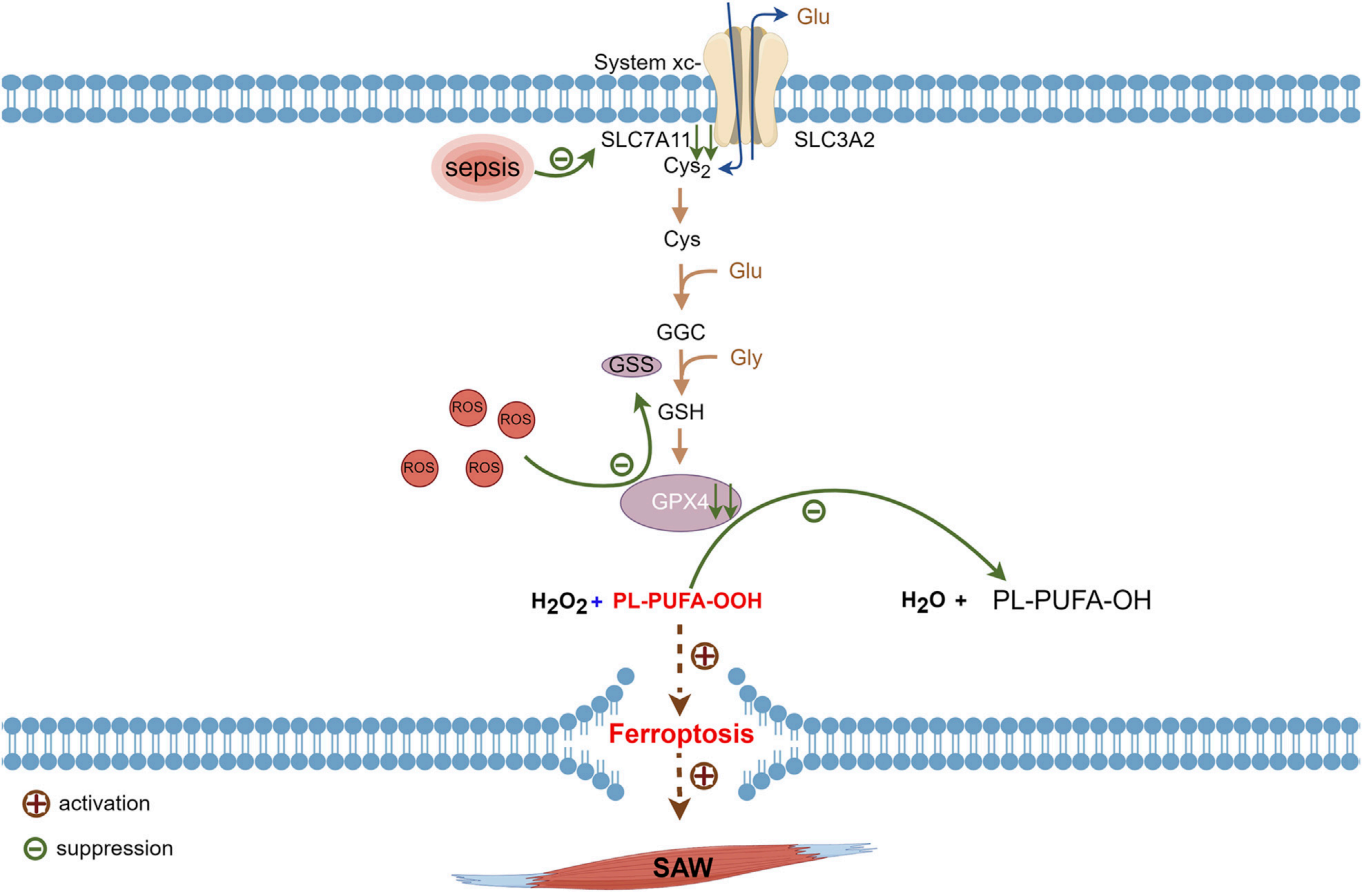
Metabolic pathways of ferroptosis associated with sepsis. Glutathione synthesized via cystine uptake of SLC7A11 is an important raw material for synthesis of GPX4, which consumes ROS generated during normal metabolism. Suppressed SLC7A11 activity during sepsis leads to downregulated GPX4 expression and impaired PL-PUFA-OOH reduction that ultimately triggers intracellular ferroptosis. GPX4, glutathione, and glutathione peroxidase 4; PL-PUFA-OOH, polyunsaturated-fatty-acid-containing-phospholipid hydroperoxides; ROS, reactive oxygen species; SLC7A11, cystine transporter solute carrier family 7 member 11.

System Xc^−^ consists of the protein subunits, SLC7A11 and heavy chain carrier family 3 member 2 (SLC3A2) that are amino acid transport proteins for cystine import and glutamate export (Dixon et al., 2014; Tang and Kroemer, 2020). Its role is to uptake cystine and excrete glutamate. Ingested cystine is reduced to cysteine then involved in glutathione synthesis (Liu et al., 2020a). Glutathione has reduced (GSH) and oxidized (GSSG) forms and GPX4 catalyzes the conversion of glutathione to GSSG. Normally, GSH plays antioxidant and protective roles by reacting with ROS (Stockwell et al., 2017). Glutathione peroxidase 4 is critical for cell survival and is a core regulator of ferroptosis, which degrades small molecule peroxides and PL-PUFA-OOH and inhibits lipid peroxidation. The knockdown and upregulation of GPX4 respectively induces and inhibits ferroptosis (Yang et al., 2014). Inhibiting SLC7A11 during sepsis leads to SXc^−^ inactivation, the prevention of cystine translocation into cells (Wang et al., 2022; Zhang et al., 2022), and reduced glutathione synthesis from glutamate and cystine. Depleted glutathione causes a decline in GPX4 synthesis, which subsequently results in a diminished ability to convert H_2_O_2_ and hydroperoxides into water and a gradual increase of hydroperoxides. Accumulated hydroperoxides further react with PUFAs on lipid membranes and lead to ferroptosis via increased lipid peroxidation of cell membranes (Yang et al., 2014; Chen et al., 2021) (Figure 3).

Sepsis is an independent risk factor for ICU-AW (Latronico et al., 2017; Mitobe et al., 2019; Bellaver et al., 2023; Takahashi et al., 2023), and SAW predominantly manifests as reduced protein synthesis coupled with increased degradation (Cohen et al., 2015). Transcription factors such as forkhead box O3 and nuclear factor kappa B required for muscle atrophy are activated during sepsis, and these transcription factors inhibit SLC7A11 expression (Zhong et al., 2023; Wu et al., 2024) which causes the inhibition of the SXc^−^-GSH-GPX4 signaling pathway (Wang et al., 2022; Zhang et al., 2022). Hence, ferroptosis facilitated by the SXc^−^-GSH-GPX4 pathway might function in the development of SAW.

#### Tumor protein 53 (p53)

**FIGURE 4:**
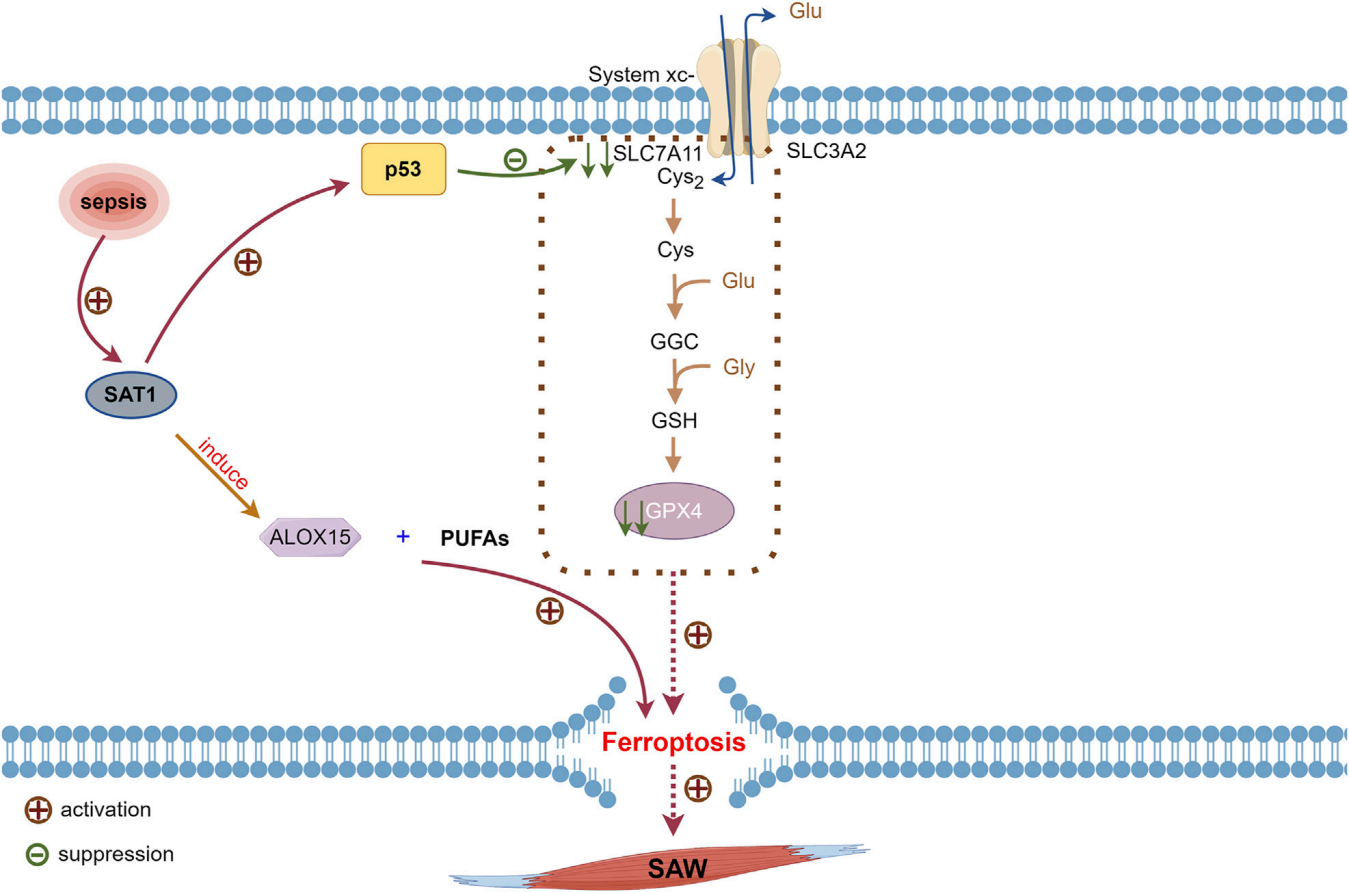
Involvement of p53 activated during sepsis on ferroptosis. Inhibition of SLC7A11 expression by p53 reduces cystine uptake, leading to decreased GPX4 synthesis. Transcriptional target of p53, SAT1, is activated in sepsis, and SAT1 induces elevation of ALOX15, which oxidizes PUFAs that promote ferroptosis. Reduced GPX4 activity inevitably leads to ferroptosis. ALOX15, arachidonate 15-lipoxygenase; GPX4, glutathione, and glutathione peroxidase 4; p53, tumor protein 53; PUFAs, polyunsaturated fatty acids; SAT1, spermidine/spermine N^1^-acetyltransferase 1.

The p53 tumor suppressor gene (Wang H. et al., 2023) is the most frequently mutated among the genes involved in human cancers (Levine, 2020). The proteins encoded by p53 control cell cycle arrest, apoptosis, and DNA repair (Hassin and Oren, 2023). Furthermore, p53 exerts its antiproliferative effects through an independent mechanism (Speidel, 2010), affects cytoplasm and almost all organelles, such as mitochondria, lysosomes, and endoplasmic reticulum (Hassin and Oren, 2023), and inhibits SLC7A11. These processes result in the inhibition of the SXc^−^-GSH-GPX4 pathway, leading to cellular ferroptosis (Jiang et al., 2015; Wang H. et al., 2023) (Figure 4).

Patients with SAW in ICU are immobilized mostly due to factors such as disease states, prevention of catheter dislodgement, and falls from beds. The expression of p53 is increased in immobilization-induced skeletal muscle atrophy (Fox et al., 2014). Spermidine/spermine N^1^-acetyltransferase 1 (SAT1) acts as a transcriptional target of p53 that is activated by inflammation; it also induces the elevated expression of arachidonate 15-lipoxygenase (ALOX15), which oxidizes PUFAs that promote ferroptosis (Ou et al., 2016; Liu et al., 2020b). Therefore, p53 and SAT1 expression might be increased in patients with SAW, which in turn induces ferroptosis. However, the exact mechanism requires further in-depth investigation.

## Summary and outlook

Sepsis-associated acquired weakness is a frequent complication in critically ill patients with sepsis that cannot be effectively treated or prevented. This type of weakness affects the quality of life of the patients and can lead to complete paralysis. Ferroptosis is a new pathway of programmed cell death in sepsis and skeletal muscle atrophy. Although there are sporadic reports suggesting that SAW is associated with ferroptosis, the mechanism remains unclear. Therefore, through this review, we described the common pathways through which ferroptosis leads to sepsis and muscle atrophy. These mechanisms potentially underpin the causative relationship between ferroptosis and SAW. However, this knowledge gap presents avenues for future investigation and serves as an impetus to guide investigators toward analyzing this situation.
